# HTLV-1 HBZ Protein Resides Exclusively in the Cytoplasm of Infected Cells in Asymptomatic Carriers and HAM/TSP Patients

**DOI:** 10.3389/fmicb.2019.00819

**Published:** 2019-04-26

**Authors:** Greta Forlani, Marco Baratella, Alessandra Tedeschi, Claudine Pique, Steve Jacobson, Roberto S. Accolla

**Affiliations:** ^1^Laboratories of General Pathology and Immunology “Giovanna Tosi,” Department of Medicine and Surgery, School of Medicine, University of Insubria, Varese, Italy; ^2^INSERM U1016, CNRS UMR 8104, Université Paris Descartes, Sorbonne Paris Cité, Institut Cochin, Paris, France; ^3^Viral Immunology Section, Neuroimmunology Branch, NINDS/NIH, Bethesda, MD, United States

**Keywords:** HTLV-1, HBZ, Tax-1, HAM/TSP, asymptomatic carriers

## Abstract

Human T cell lymphotropic virus type 1 (HTLV-1) is the causative agent of adult T cell leukemia/lymphoma (ATL) and HTLV-1-associated myelopathy/tropical spastic paraparesis (HAM/TSP) in a subset of infected subjects. Two viral proteins, Tax-1 and HTLV-1 basic leucine zipper factor (HBZ), play important roles in the pathogenesis of both diseases. We recently demonstrated that HBZ, previously considered a nuclear protein, is exclusively localized in the cytoplasm of peripheral blood mononuclear cells (PBMCs) of HAM/TSP patients. Here, the analysis of a larger panel of HAM/TSP cases confirmed that HBZ is a cytoplasmic protein, while Tax-1 preferentially localized in the cytoplasm with fewer speckle-like dots in the nucleus. More importantly, here we report for the first time that HBZ, when expressed in asymptomatic carriers (AC), is also confined in the cytoplasm. Similarly, Tax-1 was preferentially expressed in the cytoplasm in a significant proportion of AC. Interestingly, in both HAM/TSP and AC patients, the expression of HBZ and Tax-1 was rarely found in the same cell. We observed only few cases coexpressing the two oncoprotein in a very limited number of cells. In representative AC and HAM/TSP patients, cells expressing cytoplasmic HBZ were almost exclusively found in the CD4+ T cell compartment and very rarely in CD8+ T cells. Interestingly, at least in the cases analyzed, the expression of thymocite-expressed molecule involved in selection (THEMIS) is dispensable for the cytoplasmic localization of HBZ in both AC and HAM/TSP. The study of an HTLV-1-immortalized cell line established from an HAM/TSP patient confirmed HBZ as a resident cytoplasmic protein not shuttling between the cytoplasm and nucleus. These results extend our previous observation on the dichotomy of HBZ localization between HAM/TSP and ATL, pointing to the exclusive either cytoplasmic or nuclear localization in the two diseased states, respectively. Moreover, they show a rather selective expression in distinct cells of either HBZ or Tax-1. The unprecedented observation that HBZ is expressed only in the cytoplasm in AC strongly suggests a progressive modification of HBZ localization during the disease states associated to HTLV-1 infection. Future studies will clarify whether the distinct HBZ intracellular localization is a marker or a causative event of disease evolution.

## Introduction

Human T-cell leukemia virus type 1 (HTLV-1) is the first discovered human oncogenic retrovirus (Poiesz et al., 1980) and is thought to infect at least 10–15 million people worldwide. Several HTLV-1 endemic areas exist in the southern part of Japan, the Caribbean, North and South America, Central and West Africa, and foci in Middle East, Australia, and Melanesia (Gessain and Cassar, 2012). HTLV-1 induces clonal proliferation of infected cells to enhance its propagation, since it is transmitted primarily by cell-to-cell contact (Igakura et al., 2003; Pais-Correia et al., 2009; Pique and Jones, 2012; Bangham et al., 2014). It has been reported that an increased number of infected cells is correlated with a higher rate of transmission by breast-feeding (Li et al., 2004). HTLV-1 is the cause of a severe form of leukemia affecting adult CD4+ T cells (adult T-cell leukemia/lymphoma or ATL) and of a progressive neurological pathology designated HTLV-1-associated myelopathy/tropical spastic paraparesis (HAM/TSP), characterized by spastic progressive limb paralysis, sensory dysfunction, and sphincter function defects (Gessain et al., 1985; Osame et al., 1986). Similar to other retroviruses, the HTLV-1 proviral genome has structural genes, *gag*, *pol*, and *env*, coded by the plus strand of the viral genome. HTLV-1 encodes two regulatory genes, *tax* and *rex*, and three accessory genes (*p12*, *p13*, and *p30*) in the plus strand of the provirus (Matsuoka and Jeang, 2007). Another regulatory gene, the *HTLV-1 bZIP factor* (*HBZ*), is transcribed from the minus strand of the proviral genome (Larocca et al., 1989; Gaudray et al., 2002). It has been demonstrated that the viral proteins Tax-1 and HBZ play important roles in HTLV-1 infectivity as well as growth and survival of leukemic cells (Matsuoka and Jeang, 2011). Tax-1 promotes proviral transcription and tumorigenesis (Grassmann et al., 2005; Tosi et al., 2011; Forlani et al., 2013). However, Tax-1 expression is generally lost in ATL cells while HBZ transcripts are ubiquitously expressed in HTLV-1-infected cells, ATL cells, and peripheral blood mononuclear cells (PBMCs) of HTLV-1-infected individuals (Takeda et al., 2004; Taniguchi et al., 2005; Satou et al., 2006). Moreover, the amount of HBZ transcripts positively correlates with HTLV-1 proviral load (PVL) in asymptomatic carriers (AC), HAM/TSP, and ATL patients (Ma et al., 2016). Therefore, it is likely that other mechanisms are involved in the establishment and persistence of the infection, perhaps involving HBZ. Indeed, HBZ has been reported to promote growth and proliferation of leukemic cells *in vivo* and *in vitro* (Satou et al., 2006; Mitobe et al., 2015). There are three different transcriptional isoforms of HBZ: the unspliced (usHBZ) variant and two alternative spliced forms, SP1 and SP2 (Cavanagh et al., 2006; Murata et al., 2006). The SP1 form occurs more frequently than SP2 (Cavanagh et al., 2006). The sequences of SP1 and usHBZ forms are identical with the exception of the first 7 amino acids and contain 206 amino acids and 209 amino acids, respectively. Although the two protein variants exhibit similar functions (Ma et al., 2016), the spliced form is more abundant than the unspliced form and is found in almost all ATL patients (Usui et al., 2008). All the HBZ protein variants are composed by conserved functional domains: an N-terminal activation domain (AD), a central domain (CD), and a C-terminal basic ZIP domain (bZIP; Gaudray et al., 2002). HBZ displays three nuclear localization signals (NLS) responsible for its nuclear localization (Hivin et al., 2005; Zhao and Matsuoka, 2012) and two functional nuclear export signals (NES) within its N-terminal region (Mukai and Ohshima, 2011), which led us to suppose that HBZ may reside in both cytoplasm and nucleus. Most of the reported subcellular localizations, biochemical interactions, and functional aspects related to HBZ have been assessed in cells overexpressing tagged HBZ. Recently, the availability of the first reported monoclonal antibody (mAb), 4D4-F3, isolated in our laboratory, allowed us to study the expression, localization, and interaction *in vivo* of endogenous HBZ in HTLV-1-infected ACs, ATL and HAM/TSP patients (Raval et al., 2015; Baratella et al., 2017b). It was found that in chronically infected cell lines and ATL cells, endogenous HBZ interacts and colocalizes with p300 and JunD. Partial colocalization was also observed for CBP and CREB2 (Raval et al., 2015). The amount of HBZ expression in the above cells was 20- to 50-fold less than that found in HBZ-transfected cells (Raval et al., 2015; Shiohama et al., 2016). Subsequent studies have shown that HBZ localizes in different subcellular compartments in ATL and HAM/TSP. While HBZ was found in the nucleus in leukemic cells, with a speckle-like distribution (Raval et al., 2015; Baratella et al., 2017a,b), in HAM/TSP patients, we found for the first time that HBZ localized in the cytoplasm (Baratella et al., 2017b). More recently, a cytoplasmic localization of HBZ in HBZ-transfected T cells was reported (Kinosada et al., 2017), depending on the expression of THEMIS (thymocite-expressed molecule involved in selection), a T-lineage-restricted protein (Brockmeyer et al., 2011; Fu et al., 2013). Interestingly, cytoplasmic HBZ protein was almost selectively found in CD4+ T cells without relationships with CD25 expression, suggesting that CD4+ T cells were either not in rapid proliferation or not included in the classical resting regulatory T cell compartment (Baratella et al., 2017b). Consistently, it has been previously reported that HBZ-specific humoral immune response correlated with reduced CD4+ T cell activation in HAM/TSP patients (Enose-Akahata et al., 2013; Enose-Akahata et al., 2017). The distinct expression patterns of HBZ and Tax-1 in PBMCs of infected AC, ATL, and HAM/TSP patients suggest that Tax-1 and HBZ have different roles in the course of HTLV-1 pathogenesis. In the present study, the analysis of a larger panel of patients has added new relevant information on this point. PBMCs from 10 HTLV-1 AC and 10 HAM/TSP patients were analyzed. Expression and subcellular distribution of endogenous HBZ and Tax-1 proteins were assessed by confocal microscopy with the 4D4-F3 and A51-2 mAbs, respectively. Consistent with our previous findings, in HAM/TSP, HBZ protein is expressed in a discrete percentage of cells (2–10% of total PBMC), and its expression is confined in the cytoplasm. Of particular interest, we now report the unprecedented finding that HBZ is found in 6 out of 10 AC in a limited number of cells (1–4% of the total PBMC), again with an exclusive cytoplasmic localization. The number of HBZ-positive cells was higher in HAM/TSP compared to AC patients. The coexpression of Tax-1 and HBZ at the single-cell level was a very rare event both in HAM/TSP and in AC. At least for the patients analyzed, the expression of THEMIS was found not strictly required for HBZ cytoplasmic localization. Confirming our previous results, in HAM/TSP, HBZ-positive cells mainly resided in the CD4+ T cell compartment. Similar results were found in AC. These findings might shed light on a new molecular basis for a role of HBZ in the progression of HTLV-1 infection. The unprecedented observation that HBZ is expressed only in the cytoplasm in AC strongly suggests a progressive modification of HBZ localization during the disease states associated to HTLV-1 infection.

## Materials and Methods

### Ethics Statement

We obtained PBMCs from HTLV-1 asymptomatic donors and HAM/TSP patients as part of the NIH natural history protocol # 98-N-0047. All individuals gave written informed consent. The patients’ data were analyzed anonymously.

### Cells

HTLV-1-immortalized CB T cells (CB-CD4/HTLV-1; Ozden et al., 2004) were cultured in RPMI supplemented with 10% fetal calf serum (FCS) and 50 U/ml IL-2 (Sigma). PBMCs from healthy donors, HTLV-1-infected AC, or HAM/TSP patients were purified by Ficoll-Paque TM PLUS (GE-Healthcare Bio-Science, Milan, Italy) from heparinated blood. PBMCs from healthy donors were obtained from the Blood Transfusion Center, Ospedale di Circolo, Fondazione Macchi, Varese, whereas PBMCs of AC and HAM/TSP patients were isolated by Ficoll-Hypaque (Lonza) centrifugation and cryopreserved in liquid nitrogen until use.

### HTLV-1 Proviral Load Measurement

HTLV-1 PVL was measured using ddPCR (Bio-Rad) as previously described (Brunetto et al., 2014). DNA was extracted from the PBMCs and cerebro-spinal fluid (CSF) cell pellets using a DNeasy Blood and Tissue kit (Qiagen) according to the manufacturer’s instructions. DNA was digested with the restriction enzyme BamH1 (New England Biolabs) for 30 min at 37°C and diluted 1:5 with PCR-certified water. The digested, diluted DNA was mixed with both HTLV-1 *tax* and human ribonuclease P protein subunit 30 (RPP30) primers and probes and Bio-Rad 2× Supermix and then emulsified with droplet generator oil using a QX-100 droplet generator according to the manufacturer’s instructions (Bio-Rad). The following primers and probe were used to amplify and detect a 154-base-pair region of HTLV-1 *tax*: ddPCR HTLV-1 *tax* F: 5′-CTTATTTGGACATTTACCGATG-3′; ddPCR HTLV-1 *tax* R: 5′-TGAGGCCGTGTGAGAGTAGA-3′; ddPCR HTLV-1 *tax* probe: 6FAM-TGATTTCCGGGCCCTGC-MGBNFQ. The droplets were then transferred to a 96-well reaction plate (Eppendorf) and heat-sealed with pierceable sealing foil sheets (Thermo Fisher Scientific). The duplex PCR amplification was performed in this sealed 96-well plate using a GeneAmp 9700 thermocycler (Applied Biosystems). Following PCR amplification, the 96-well plate was transferred to a QX100 droplet reader (Bio-Rad). For PVL calculation, QuantaSoft software version 1.3.2.0 (Bio-Rad) was used to quantify the copies per microliter of each queried target per well. All samples were tested in duplicate, unless otherwise specified, and PVL is reported as the average of the two measurements. The PVL was calculated using the following formula: PVL = [quantity of HTLV-1 *tax*/(quantity of RPP30/2)] × 100%.

### Treatments

CB-CD4/HTLV1 cells cultured on glass coverslips precoated with poly-L-lysine were incubated with 20 nM leptomycin B (LMB; Sigma) or the vehicle methanol for 3 h at 37°C, 5% CO_2_. Cells were then washed and processed for confocal microscopy analysis by using the following antibodies: anti-HBZ 4D4-F3 mAb, anti-Tax-1 mAb (clone 168 A51-2 from the NIH AIDS Research and Reference Reagent Program), and anti-RelA rabbit polyclonal antibody (Santa Cruz Biotechnology, CA, United States), followed by the secondary antibodies as specified in the figure legends.

### Immunofluorescence, Flow Cytometry, and Confocal Microscopy

Peripheral blood mononuclear cells from AC, HAM/TSP patients, or normal control individuals, after rapid thawing, were washed with warm RPMI medium and immediately processed for immunofluorescence and flow cytometry analysis or for confocal microscopy, as described (Forlani et al., 2016b). For flow cytometry, the following reagents were used: mouse anti-human HLA class I (clone B9.12); mouse anti-human HLA class II DR (clone D1.12), both revealed by FITC-labeled rabbit anti-mouse IgG F(ab’)2 antiserum (Sigma, Milan, Italy); FITC mouse anti-human CD3 (clone (UCHT1, BD Pharmingen); FITC mouse anti-human CD4 (clone RPA-T4, BD Pharmingen); PE-Cy5 mouse anti-human CD8a (clone RPA-T8; eBioscience, Milan, Italy); PE mouse anti-human CD16 (clone B73.1, eBioscience, Milan, Italy); FITC mouse anti-human CD19 (clone HIB19, BD Pharmingen); and phycoerythrin (PE) mouse anti-human CD25 (clone M-A251, BD Pharmingen). For confocal microscopy, cells were cultured on glass coverslips precoated with poly-L-lysine (0.2 g/ml, Sigma) for 5 h. The cells were then washed with 1× Pipes-Hepes-EGTA-MgSO4 (PHEM) buffer, pH 6.9 (60 mM PIPES, 25 mM 4-(2-hydroxyethyl)-1-piperazineethanesulfonic acid (HEPES), 10 mM ethylene glycol-bis(beta-aminoethyl ether)-N, N, N′, N′-tetraacetic acid (EGTA), and 2 mM MgCl_2_), three times, fixed in methanol 7 min at −20°C, and blocked with 1% bovine serum albumin (BSA) in 1× PHEM for 1 h at room temperature (RT). Cells were then stained overnight with anti-HBZ 4D4-F3 mAb, anti-Tax-1 mAb (clone 168 A51-2 from the NIH AIDS Research and Reference Reagent Program), anti-vimentin rabbit polyclonal antibody (Santa Cruz Biotechnology, CA, United States), rabbit anti-CD4 mAb (clone EPR6855, ABCAM), and rabbit anti-THEMIS mAb (clone EPR7353, ABCAM), diluted in PHEM buffer containing 0.5% BSA. The slides were then washed five times with cold 1× PHEM and incubated in the dark for 2 h at RT with the following secondary antibodies from Life Technology (Waltham, MA, United States): goat anti-mouse IgG1 coupled to Alexa Fluor 546 to detect HBZ, goat anti-mouse IgG2a conjugated to Alexa Fluor 488 to detect Tax-1, and goat anti-rabbit IgG conjugated to Alexa Fluor 488 or to Alexa Fluor 546 to detect vimentin, CD4, or THEMIS. For co-staining with directly labeled antibodies, after extensive washing with 1× PHEM, anti-CD8 rabbit mAb directly conjugated to Alexa Fluor 647 (clone EP1150Y, ABCAM) and mouse anti-human CD25 mAb directly conjugated to Alexa Fluor 488 (clone BC96, BioLegend) were added after the indirect immunofluorescence for 2 h at RT. Similarly, after indirect immunofluorescence, the nuclei were stained by incubating the cells with DRAQ5 Fluorescent Probe (Thermo Fisher Scientific, Waltham, MA, United States) for 30 min at RT. CIB-CD4/HTLV1 cells were cultured on glass coverslips precoated with poly-L-lysine for 5 h and processed for confocal microscopy analysis as described above.

After washing, the slides were mounted on coverslips with the Fluor Save reagent [Calbiochem, Vimodrone (MI), Italy] and examined by a confocal laser scanning microscope (Leica TCS SP5; HCX PL APO objective lenses, 63× original magnification, numerical aperture 1.25). Images were acquired and analyzed by LAS AF Lite Image (Leica Microsystems, Milan, Italy) and/or Fiji (ImageJ) software.

### Preparation of Nuclear and Cytoplasmic Extracts and Immunoprecipitation Procedures

Endogenous HBZ protein was precipitated from both the nuclear and cytoplasmic protein fractions prepared from 8 × 10^6^ CD-CD4/HTLV-1 cells, using an NE-PER Nuclear Cytoplasmic Extraction Reagent kit (Thermo Fisher Scientific) according to the manufacturer’s instruction. Briefly, cells were washed twice with cold phosphate buffer saline (PBS) and centrifuged at 500*g* for 3 min. The cell pellet was suspended in 200 μl of cytoplasmic extraction reagent I (CERI), supplemented with 0.1% protease inhibitor cocktail (Sigma), by vortexing. The suspension was incubated on ice for 10 min followed by the addition of 11 μl of a second cytoplasmic extraction reagent II (CERII), vortexed for 5 s, incubated on ice for 1 min, and centrifuged for 5 min at 16,000*g*. The supernatant fraction (cytoplasmic extract) was transferred to a pre-chilled tube. The insoluble pellet fraction, which contains crude nuclei, was resuspended in 100 μl of nuclear extraction reagent (NER), supplemented with 0.1% protease inhibitor cocktail (Sigma), by vortexing for 15 s, incubated on ice for 40 min, and then centrifuged for 10 min at 16,000*g*. Both the nuclear and cytoplasmic protein extracts were used for the subsequent immunoprecipitation experiments.

After preclearing with protein A/G-Sepharose beads, the nuclear and cytoplasmic extracts were incubated with anti-HBZ 4D4-F3 mAb for 1 h on ice and then immunoprecipitated with protein A/G-Sepharose beads overnight at 4°C, under rotation. The precipitated proteins were resolved on 9% sodium dodecyl sulfate-polyacrilamide gel electrophoresis (SDS-PAGE) and analyzed by Western blotting with the anti-HBZ 4D4-F3 mAb, followed by Mouse True Blot ULTRA-anti mouse Ig HRP (e Bioscience). This specific secondary antibody was used to avoid the detection of both heavy and light chains in the precipitated proteins. Ten percent of the nuclear and cytoplasmic protein extracts were analyzed by Western blotting with anti Nup98 (Cell Signaling Technology) and anti-β-tubulin (Sigma) mAbs to assess the purity of nuclear and cytoplasmic protein extracts, respectively.

## Results

### PBMCs of HTLV-1-Positive Asymptomatic Carriers Express HBZ Protein in the Cytoplasm

**FIGURE 1:**
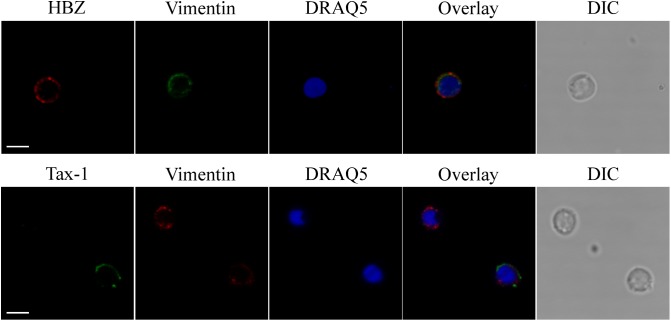
Endogenous HBZ localized in the cytoplasm whereas Tax-1 was distributed both in the nucleus and in the cytoplasm in peripheral blood mononuclear cells (PBMCs) of representative AC PH1307. PBMCs of representative AC PH1307 patient were stained with the 4D4-F3 anti-HBZ monoclonal antibody (mAb) followed by Alexa Fluor 546-conjugated goat anti-mouse IgG1 antibody (red, upper panels) and with the A51-2 anti-Tax-1 mAb followed by Alexa Fluor 488-conjugated goat-anti-mouse IgG2a antibody (green, lower panels) and analyzed by confocal microscopy. Specific counterstaining of nucleus or cytoplasmic compartments was performed by using DRAQ5 fluorescence probe to detect the nucleus (blue) and anti-vimentin rabbit polyclonal antibody followed by goat anti-rabbit IgG conjugated to Alexa Fluor 488 (green, upper panels) or to Alexa Fluor 546 (red, lower panels) to stain the cytoplasmic compartment. DIC represents the differential interference contrast image. At least 300 cells were analyzed. One representative image of single confocal section for HBZ or Tax-1 staining is shown. All scale bars are 5 μm.

By analyzing the subcellular distribution of endogenous HBZ in PBMCs of four HAM/TSP patients, we previously demonstrated for the first time that HBZ is a cytoplasmic protein expressed in a discrete percentage, up to 11%, of the cells. Unlike HAM/TSP, we were unable to detect HBZ-positive cells in PBMCs of four AC (Baratella et al., 2017a,b). To better understand the role of HTLV-1 HBZ protein in the progression of HTLV-1-associated diseases, we extended our previous study to include more HAM/TSP and AC patients. We first analyzed by immunofluorescence and confocal microscopy the subcellular localization of both endogenous HBZ and Tax-1 oncoproteins in PBMCs isolated from 10 HTLV-1-positive AC (Table 1). Interestingly, although PBMC from four ACs were negative for HBZ expression, six ACs clearly expressed HBZ protein in 1–4% of their PBMCs (Table 1). More importantly, HBZ was found localized exclusively in the cytoplasm (Figure 1, HBZ). Parallel staining with vimentin, a cytoplasmic marker, and DRAQ5, a nuclear marker, confirmed HBZ cytoplasmic localization without detectable nuclear colocalization (Figure 1, upper panels). Like HAM/TSP patients, in PBMCs from AC, cytoplasmic HBZ appeared distributed in dots, sometimes dispersed all over the cytoplasm or concentrated in restricted areas. The expression and the subcellular distribution of Tax-1 were then analyzed. Tax-1 was expressed in 70% of ACs in a discrete proportion of cells (between 2 and 9% of total PBMCs). Tax-1 was preferentially localized in the cytoplasm, with fewer speckle-like dots in the nucleus (Figure 1, Tax-1). Co-staining with vimentin and DRAQ5 showed the Tax-1 prominent cytoplasmic localization (Figure 1, bottom panels).

**Table 1:** Percent distribution of HBZ+ and Tax-1+ PBMCs in HTLV-associated pathologies.

Patient	Proviral load	Pathology	Total HBZ+ cells (%)	Total Tax-1+ cells (%)	HBZ+ Tax-1+ cells (%)
PH70	3.26	AC^∗^	1	0	0
PH928	1.02	AC	0	3	0
PH1152	6.43	AC	0	2	0
PH1186	0	AC	0	4	0
PH1307	8	AC	3	4	0
PH1320	0	AC	2	9	0.2
PH1339	1.1	AC	4	7	1
PH1443	2.81	AC	1	2	0
PH2113	0.58	AC	0	0	0
PH2116	19.16	AC	2	0	0
PH667	8.84	HAM/TSP	0	11	0
PH1026	27.21	HAM/TSP	6	3	0
PH1205	9.99	HAM/TSP	7	2	0
PH1216	0.74	HAM/TSP	8	15	1
PH1419	6.69	HAM/TSP	4	7	0
PH1509	5.09	HAM/TSP	10	2	0
PH1510	16.59	HAM/TSP	2	4	0
PH2163	20.13	HAM/TSP	1	12	0
PH2176	27.44	HAM/TSP	1	5	0
PH2262	20.44	HAM/TSP	2	3	0

*^∗^AC, asymptomatic carriers.*

**FIGURE 2:**
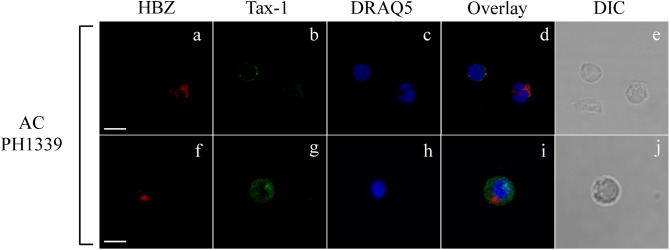
Coexpression of HBZ and Tax-1 in the same cell is a rare event in asymptomatic carriers. PBMCs of representative AC PH1339 patient were co-stained with the 4D4-F3 anti-HBZ mAb followed by Alexa Fluor 546-conjugated goat anti-mouse IgG1 antibody (red, **a,f,d,i**) and with the A51-2 anti-Tax-1 mAb followed by Alexa Fluor 488-conjugated goat-anti-mouse IgG2a antibody (green, **b,g,d,i**) and analyzed by confocal microscopy. DRAQ5 fluorescence probe was used to detect the nucleus **(c,h,d,i)**. DIC represents the differential interference contrast image **(e,j)**. At least 300 cells were analyzed. In the top panel, one representative image of single confocal section is shown; in the bottom panel, one representative image derived from the same sample and obtained from the sum of all z-stacks is shown. All scale bars are 5 μm.

Interestingly, expression of both HBZ and Tax-1 was found in PBMCs of only four ACs, but in two ACs coexpression of HBZ and Tax-1 within the same cell was detected in a small proportion of cells (0.2 or 1% of total PBMCs; Table 1, PH1320 and PH1339, respectively), indicating that, in AC, the coexpression of the two viral proteins in the same cell is a rare event. A clear example was the PH1339 AC, whose PBMCs were 4 and 7% positive for HBZ and Tax-1, respectively (Figure 2a,b, respectively and Figure 2d) while only 1% coexpressed the two viral proteins (Figure 2f,g, respectively). In coexpressing cells, HBZ did not colocalize with Tax-1 (Figure 2i). Moreover, HBZ was confined in a specific region of the cytoplasm (Figure 2f), while Tax-1 was observed in the nucleus and in the cytoplasm surrounding the HBZ-positive staining (Figure 2g). DRAQ5 was used to stain the nuclei (Figure 2c,h). Differential interference contrast (DIC) images of the cells are shown (Figure 2e,j).

In addition, in AC PH70 and PH2116, HBZ was expressed in 1 and 2% of total PBMC, respectively, while Tax-1 was not detected. On the contrary, in AC PH928, PH1152, and PH1186, HBZ was undetectable, while Tax-1 was found in 3, 2, and 4% of total PBMCs, respectively. We were unable to detect both HBZ and Tax-1 in patient PH2113 (Table 1). Collectively, these findings demonstrate for the first time that HBZ protein has a cytoplasmic localization in AC and this localization is maintained in HAM/TSP patients.

### HAM/TSP Pathology Is Characterized by a Relevant Increase in Number of HBZ-Positive Cells With an Exclusive Cytoplasmic Localization

**FIGURE 3:**
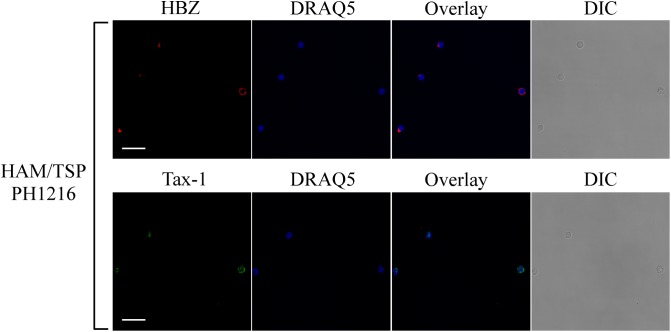
Subcellular localization of endogenous HBZ and Tax-1 in PBMCs of representative HAM/TSP PH1216 patient. (Top panels) PBMCs of representative HAM/TSP patient PH1216 were stained with the 4D4-F3 anti-HBZ mAb followed by Alexa Fluor 546-conjugated goat anti-mouse IgG1 antibody (red) and (Bottom panels) with the A51-2 anti-Tax-1 mAb followed by Alexa Fluor 488-conjugated goat-anti-mouse IgG2a antibody (green) and analyzed by confocal microscopy. Nucleus was stained with DRAQ5. DIC represents the differential interference contrast image. At least 300 cells were analyzed; two representative fields for HBZ or Tax-1 staining are shown. All scale bars are 5 μm.

To expand our previous studies in HAM/TSP patients, PBMCs of 10 additional patients were investigated by immunofluorescence and confocal microscopy. Consistent with our previous findings, nine HAM/TSP patients expressed HBZ with a percentage of HBZ-positive cells ranging between 1 and 10% (Table 1). Interestingly, the number of HBZ-positive cells was higher in HAM/TSP patients as compared to AC. As clearly shown for a representative patient PH1216 having 8% of HBZ-positive cells, the viral protein localized in the cytoplasm with a speckled-like distribution dispersed along the cytoplasm or accumulated in a discrete and limited region of it (Figure 3, HBZ). Parallel staining with DRAQ5 confirmed the HBZ cytoplasmic distribution (Figure 3, top panels, overlay). Tax-1 was expressed in all HAM/TSP patients with a percentage of Tax-1-positive cells ranging between 2 and 15% of total PBMCs depending on the patient analyzed (Table 1). Patient PH667 did not show any positivity for HBZ, although it expressed Tax-1 in a considerable number of cells (11%; Table 1).

**FIGURE 4:**
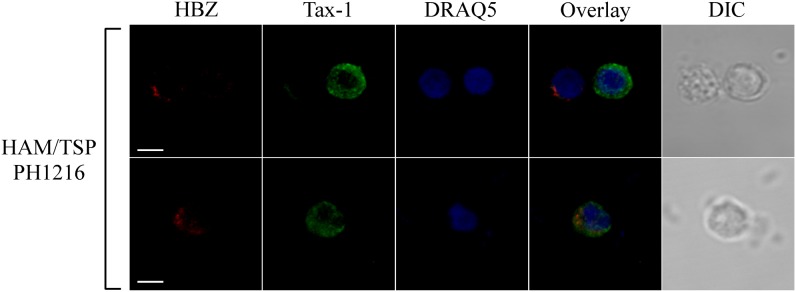
HBZ and Tax-1 were coexpressed at the single-cell level in 1% of total PBMCs of HAM/TSP representative patient PH1216. PBMCs of representative HAM/TSP PH1216 patient were co-stained with the 4D4-F3 anti-HBZ mAb followed by Alexa Fluor 546-conjugated goat anti-mouse IgG1 antibody (red) and with the A51-2 anti-Tax-1 mAb followed by Alexa Fluor 488-conjugated goat-anti-mouse IgG2a antibody (green) and analyzed by confocal microscopy. DRAQ5 fluorescence probe was used to detect the nucleus. DIC represents the differential interference contrast image. At least 300 cells were analyzed; representative images for HBZ and Tax-1 co-staining are shown. All scale bars are 5 μm.

Tax-1 was expressed mostly as discrete dots localized preferentially in the cytoplasm with fewer dots in the nucleus (representative patient PH1216; Figure 3, Tax-1). In this patient, expressing the highest number of HBZ and Tax-1 positive cells, 8 and 15%, respectively (see Table 1), most cells expressed either HBZ or Tax-1 (Figure 4, upper panels), with only 1% of the cells coexpressing the two viral proteins. In some cells, HBZ partially colocalized with Tax-1 in the cytoplasm (Figure 4, bottom panels, overlay).

Thus, as we observed for AC, in HAM/TSP patients, coexpression of HBZ and Tax-1 oncoproteins is a rare event. Importantly, the cumulatively higher number of HBZ- and Tax-1-expressing cells in HAM/TSP patients compared to HTLV-1-infected AC strongly suggests that evolution toward the neuroinflammatory disease modifies only the number of HBZ- and Tax-1-positive cells but not the HBZ- or the Tax-1-specific intracellular localization. Moreover, the higher number of HBZ- and Tax-1-expressing cells in HAM/TSP compared to AC correlated generally, but not always, with higher PVL (Table 1).

### CD4+ T Cell Subpopulation Represents the Majority of HBZ-Positive Cells in Asymptomatic Carriers and in HAM/TSP Patients

We previously demonstrated that in HAM/TSP patients, cytoplasmic HBZ is almost exclusively found in CD4+ T cells not coexpressing the CD25 marker (Baratella et al., 2017b). In order to identify the cell subpopulations expressing the cytoplasmic HBZ protein in AC patients and extend the analysis to the new HAM/TSP patients, we first analyzed by immunofluorescence and flow cytometry the cell surface phenotype of PBMCs of all AC and HAM/TSP patients.

**FIGURE 5:**
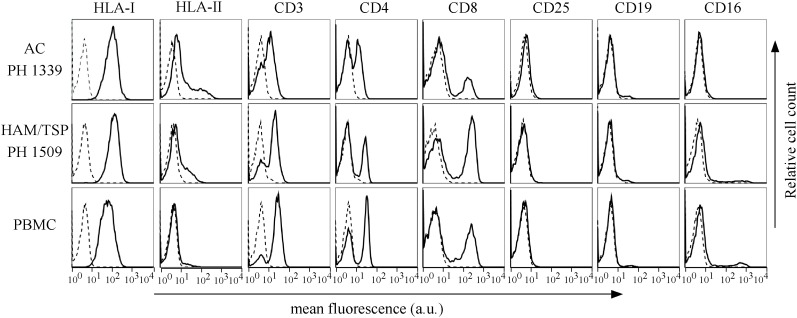
Expression of cells surface markers in PBMCs of AC PH1339, HAM/TSP patient PH1509, and healthy control. The expression of HLA class I, HLA class II DR, CD3, CD4, CD8, CD25, CD19, and CD16 surface molecules on PBMCs from AC PH1339, HAM/TSP patient PH1509, and a healthy control was assessed by immunofluorescence and flow cytometry with antibodies specific for the various markers. Results are expressed as relative number of cells (ordinate) versus the mean intensity of fluorescence in arbitrary units (abscissa). In each histogram, negative controls, obtained by staining the cells with an appropriate isotype-matched antibody, are depicted as a dashed line.

Peripheral blood mononuclear cells of AC and HAM/TSP patients displayed a phenotype similar to that of PBMCs from healthy donors (Figure 5, representative AC PH1339 and representative HAM/TSP PH1509). PH1339 expressed CD3, CD4, and CD8 markers in 61, 44, and 18% of PBMCs, respectively. CD3 and CD4 T cell markers were expressed in a comparable number of cells in patient HAM/TSP PH1509 (70 and 33%, respectively) while cells expressing the CD8 marker were increased to 40%. In the sample analyzed, the CD25 marker was undetectable. The CD19-positive B cells were present in almost equal proportions of PH1307 (4.5%), PH1419 (4.7%), and normal (3.4%) PBMCs. NK cells, as assessed by the CD16 marker, were not detected in PH1307, whereas they represented 11.4 and 10% of PH1419 and normal PBMCs, respectively. HLA-I was expressed in 100% of PBMCs of the patients and the normal donor, and HLA class II was expressed in 17, 12, and 9% of PH1339, PH1509, and normal PBMCs, respectively. Subsequently, we co-stained PBMCs of AC and HAM/TSP patients for either CD4 or CD8 and HBZ, and we analyzed them by confocal microscopy. Representative data obtained from PBMCs of PH1339 AC and PH1509 HAM/TSP patient revealed a higher number of HBZ-positive cells(7 and 10%, respectively).

**FIGURE 6:**
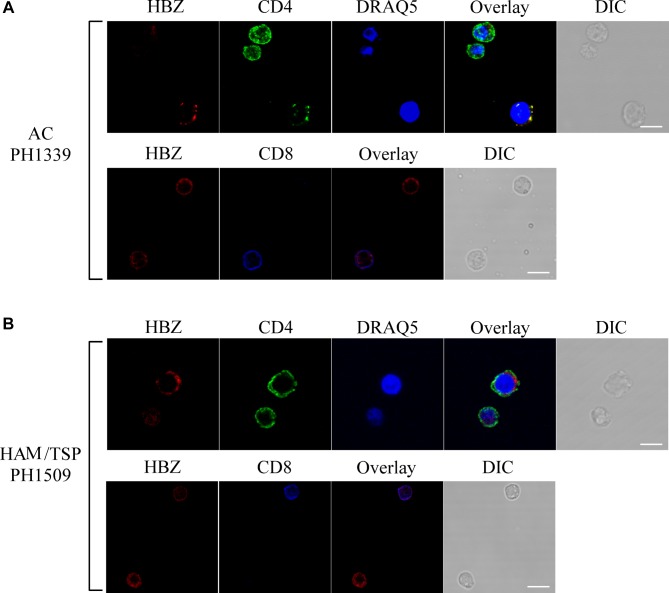
HBZ is preferentially expressed in CD4+ T cells of AC PH1339 and HAM/TSP patient PH1509. Confocal microscopy analysis of PBMCs from AC patient PH1339 and HAM/TSP patient PH1509. **(A)** Upper panels: PBMC of patient AC PH1339 were co-stained with the 4D4-F3 anti-HBZ mAb followed by Alexa Fluor 546-conjugated goat anti-mouse IgG1 antibody (red) and with the anti-CD4 mAb followed by Alexa Fluor 488-conjugated goat-anti-rabbit IgG antibody (green). Lower panels: co-staining with the 4D4-F3 anti-HBZ mAb followed by Alexa Fluor 546-conjugated goat anti-mouse IgG1 antibody (red) and with the anti-CD8 rabbit mAb directly conjugated to Alexa Fluor 647 (blue). **(B)** PBMCs of patient HAM/TSP PH1509 were co-stained with 4D4-F3 anti-HBZ mAb and either anti-CD4 mAb (upper panels) or anti-CD8 mAb (lower panels) followed by specific secondary antibodies staining as described in panel **(A)**. DIC represents the differential interference contrast image. At least 200 cells were analyzed; representative images derived from each sample are shown. All scale bars are 5 μm.

Almost all HBZ-positive cells expressed CD4 (Figure 6A, upper panels). Conversely, HBZ-expressing cells were very rarely detected in the CD8+ T cell compartment (Figure 6A, lower panels).

Similarly to AC patients and confirming our previous findings (Baratella et al., 2017b) in HAM/TSP PH1509 patient, cells expressing cytoplasmic HBZ were almost exclusively found in the CD4+ T cells compartment (Figure 6B, top panels) and very rarely, less than 1%, in CD8+ T cells (Figure 6B, lower panels). From the above data, we conclude that in AC and HAM/TSP patients, cytoplasmic HBZ is almost exclusively expressed in CD4+/CD25- T cells.

### The Expression of THEMIS Is Dispensable for the Cytoplasmic Localization of HBZ in Asymptomatic Carriers and HAM/TSP Patients

Recently, it has been reported that the HBZ-transfected Jurkat T cell line may partially segregate HBZ in the cytoplasm as a result of interaction with THEMIS (Kinosada et al., 2017), a molecule involved in thymocyte selection and T cell receptor (TCR) signaling.

**FIGURE 7:**
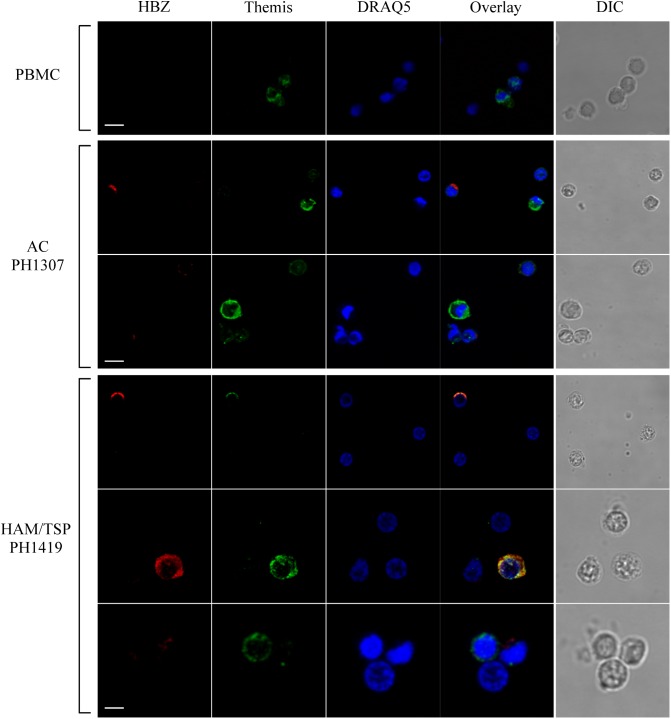
Subcellular localization of endogenous HBZ and THEMIS in PBMC of AC, HAM/TSP patients, and healthy donor. Co-staining of PBMCs of representative AC PH1307 and HAM/TSP 1419 and healthy donor (PBMC) was performed using antibodies against HBZ (red) and THEMIS (green) followed by Alexa Fluor 546-conjugated goat anti-mouse IgG1 antibody and by Alexa Fluor 488-conjugated goat-anti-rabbit IgG antibody, respectively. DIC represents the differential interference contrast image. At least 200 cells were analyzed; two representative images derived from each sample are shown. All scale bars are 5 μm.

To assess whether HBZ colocalizes with THEMIS in PBMCs, we analyzed by immunofluorescence and confocal microscopy the expression and subcellular localization of both HBZ and THEMIS in PBMCs of AC and HAM/TSP patients. Figure 7 shows the results obtained in PBMCs of PH1307 and PH1419, representative of an AC and a HAM/TSP patient, respectively. As control, PBMCs from non-infected individuals were also analyzed. As expected, in PBMCs of a healthy donor, THEMIS was expressed in the cytoplasm of a discrete percentage of cells (Figure 7, PBMC, Themis). The co-staining with DRAQ5 nuclear marker confirmed THEMIS as a cytoplasmic resident protein (Figure 7, PBMC, overlay).

Interestingly, in PBMCs of PH1307 AC, coexpression of HBZ and THEMIS could be found in cells expressing both proteins at low level (Figure 7, AC PH1307, bottom panels). On the other hand, highly expressing THEMIS-positive cells (Figure 7, AC PH1307, top panels, Themis) usually did not show coexpression of HBZ (Figure 7, AC PH1307, top panels, THEMIS and overlay). Similarly, highly expressing HBZ cells can be found that do not express THEMIS (Figure 7, AC PH1307, top panels, HBZ and overlay).

Unlike AC, in PBMCs of HAM/TSP patient PH1419, the majority of HBZ-positive cells (Figure 7, HAM/TSP PH1419, top and middle panels, HBZ) were included in the highly expressing THEMIS (Figure 7, HAM/TSP PH1419, top and middle panels, Themis) and the two proteins partially colocalized in the cytoplasm (Figure 7, HAM/TSP PH1419, top and middle panels, overlay). Nevertheless, we found cells expressing either THEMIS or HBZ alone (Figure 7, HAM/TSP PH1419, bottom panels). Collectively, these findings suggest that colocalization of HBZ and THEMIS is observed, but the cytoplasmic expression of THEMIS is not required for the cytoplasmic expression of HBZ, particularly in HAM/TSP patients.

### An IL-2-Dependent Cell Line From a HAM/TSP Patient Further Demonstrates HBZ as a Cytoplasmic Protein Unable to Shuttle Between Cytoplasm and Nucleus

**FIGURE 8:**
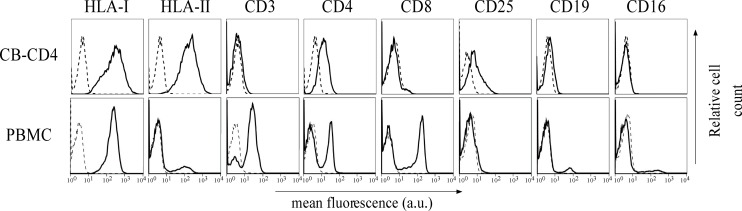
Expression of cell surface markers in the CB-CD4/HTLV-1 cell line. The expression of HLA class I, HLA class II DR, CD3, CD4, CD8, CD25, CD19, and CD16 surface molecules on CB-CD4 HAM/TSP cells and healthy control was assessed by immunofluorescence and flow cytometry with antibodies specific for the markers indicated on the top of the panels. Results are expressed as relative number of cells (ordinate) versus the mean intensity of fluorescence in arbitrary units (abscissa). In each histogram, negative controls, obtained by staining the cells with an appropriate isotype-matched antibody, are depicted as a dashed line.

**FIGURE 9:**
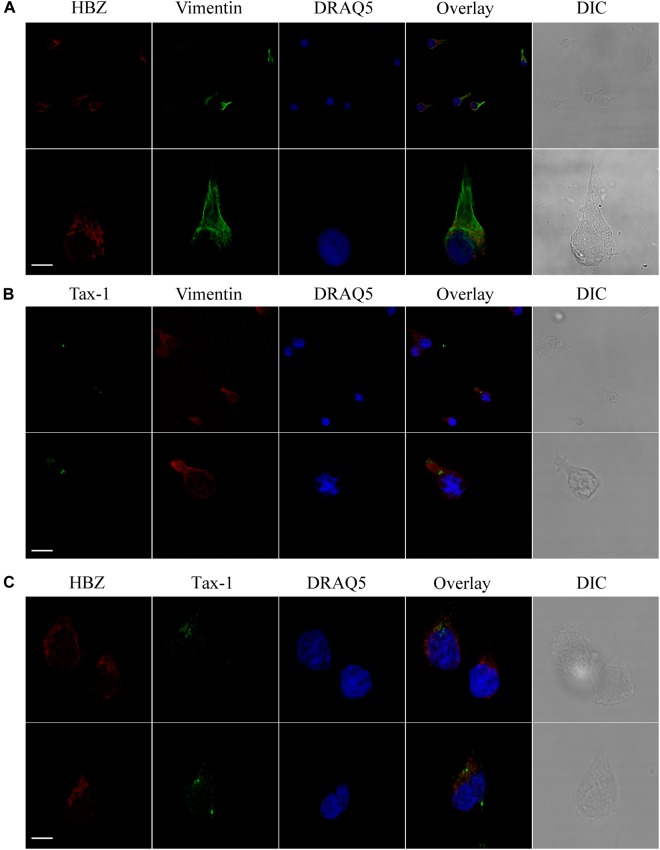
In CB-CD4/HTLV-1 cells, HBZ and Tax-1 reside in different compartments of the cytoplasm. **(A)** CB-CD4/HTLV-1 cells were stained with the 4D4-F3 anti-HBZ mAb followed by Alexa Fluor 546-conjugated goat anti-mouse IgG1 antibody (red), with DRAQ5 to detect the nucleus (blue) and with anti-vimentin rabbit polyclonal antibody followed by goat anti-rabbit IgG conjugated to Alexa Fluor 488 (green) to detect the cytoplasmic compartment. DIC represents the differential interference contrast image. Three representative images for HBZ staining are shown. **(B)** CB-CD4/HTLV-1 cells were stained with the A51-2 anti-Tax-1 mAb followed by Alexa Fluor 488-conjugated goat-anti-mouse IgG2a antibody (green), with DRAQ5 to detect the nucleus (blue) and with anti-vimentin rabbit polyclonal antibody followed by goat anti-rabbit IgG conjugated to Alexa Fluor 546 (red) to detect the cytoplasmic compartment. DIC represents the differential interference contrast image. Two representative images for Tax-1 staining are shown. **(C)** CB-CD4/HTLV-1 T cells were co-stained with the 4D4-F3 anti-HBZ mAb followed by Alexa Fluor 546-conjugated goat anti-mouse IgG1 antibody (red) and with the A51-2 anti-Tax-1 mAb followed by Alexa Fluor 488-conjugated goat-anti-mouse IgG2a antibody (green) and analyzed by confocal microscopy. DRAQ5 was used to detect the nucleus. DIC represents the differential interference contrast image. Two representative images are shown. All scale bars are 5 μm.

**FIGURE 10:**
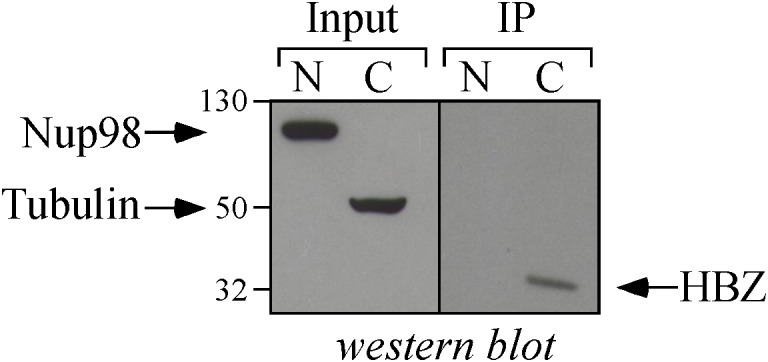
Biochemical assessment of the cytoplasmic localization of HBZ in CB-CD4/HTLV-1 cells. Nuclear (N) and cytoplasmic (C) protein extracts obtained from CB-CD4/HTLV-1 cells (8 × 10^6^ cells) were immunoprecipitated with an anti-HBZ 4D4-F3 mAb (IP) and analyzed for the presence of HBZ by Western blotting using the same antibody (HBZ). Ten percent of the nuclear and cytoplasmic protein extracts were analyzed for the expression of Nucleoporin 98 (NUP98) and β-tubulin (Tubulin) by Western blotting (input).

**FIGURE 11:**
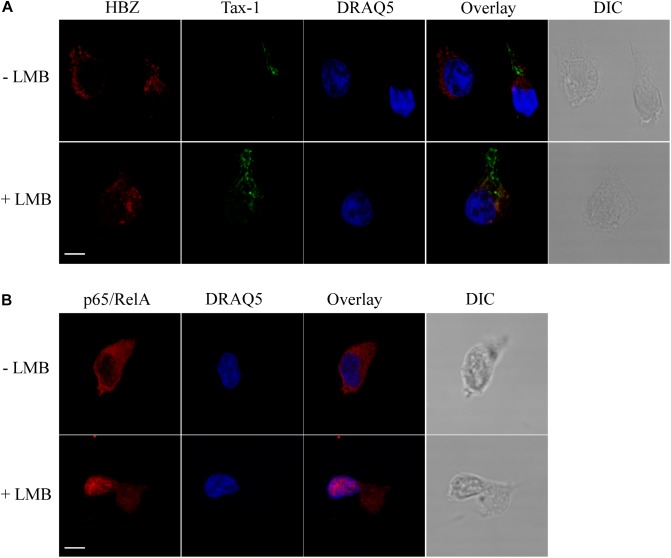
HBZ is a resident cytoplasmic protein and does not shuttle to the nucleus in CB-CD4/HTLV-1 cells. **(A)** Cells were either treated (+LMB) or not treated (-LMB) with LMB, an inhibitor of nuclear export, before fixing and co-staining with the anti-HBZ 4D4-F3 mAb followed by Alexa Fluor 546-conjugated goat anti-mouse IgG1antibody (red) and A51-2 anti-Tax-1 mAb followed by Alexa Fluor 488-conjugated goat-anti-mouse IgG2a antibody (green). DRAQ5 was used to detect the nucleus (blue). One representative image is shown for each sample. **(B)** As control of inhibition of nuclear export by LMB, treated (+LMB) and untreated (-LMB) cells were fixed and stained with antibodies against p65/RelA followed by Alexa Fluor 546-conjugated goat-anti-rabbit secondary antibody (red) and analyzed by confocal microscopy. Nucleus was stained with DRAQ5 (blue). DIC represents the differential interference contrast image. One representative image is shown for each sample. All scale bars are 5 μm.

As shown above, HBZ expression in HTLV-1-infected asymptomatic patients shows a cytoplasmic localization that is further increased both in intensity and in number of positive cells in HAM/TSP patients. To further investigate the biological basis of this peculiar subcellular distribution of HBZ compared with the prominent nuclear localization found in ATL cells, we took advantage of an IL-2-dependent HTLV-1-immortalized T cell line, designated CB-CD4/HTLV-1 established from a HAM/TSP patient (Ozden et al., 2004). Interestingly, the cell surface phenotype of CB-CD4/HTLV-1 showed that these cells express CD4, CD25, and HLA-II markers (Figure 8, upper panels) but lack the cell surface expression of CD3. Absence of CD3 expression in HTLV-1-infected cells is not unprecedented since it has been observed also in other cell lines (Raval et al., 2015). As observed in fresh PBMCs of HAM/TSP patients, HBZ was found to be localized in the cytoplasm of CB-CD4/HTLV-1 cells (Figure 9A, HBZ). Parallel staining with DRAQ5 or with vimentin confirmed the exclusive HBZ cytoplasmic localization with a similar speckle-like distribution as the one observed in PBMCs of HAM/TSP patients (Figure 9A, overlay). While virtually all cells expressed HBZ, only 20% of them expressed Tax-1. Interestingly, Tax-1 was mainly localized in the cytoplasm with a dotted-like distribution (Figure 9B, Tax-1). Parallel staining with DRAQ5 and vimentin revealed that Tax-1 is concentrated in a specific cytoplasmic area (Figure 9B, overlay). Furthermore, we observed that Tax-1 distribution did not overlap with that of HBZ, suggesting that the two proteins localized in different regions of the cytoplasmic compartment (Figure 9C, overlay). Altogether these localization studies suggested that HBZ resided exclusively in the cytoplasm of CB-CD4/HTLV-1 cells. To further confirm the cytoplasmic localization of HBZ, nuclear and cytoplasmic fractions of CB-CD4/HTLV-1 cells were immunoprecipitated with the 4D4-F3 anti-HBZ mAb, with the resulting immunoprecipitate run in SDS-PAGE and analyzed by Western blotting with the same antibody. As shown in Figure 10, HBZ protein was found only in the cytoplasmic fraction (C) of CB-CD4/HTLV-1 cells (Figure 10, IP, HBZ). Nucleoporin 98 (Nup98) and Tubulin protein expression was also analyzed (Figure 10, input) to verify the purity of nuclear (N) and cytoplasmic (C) protein extracts. To assess whether, in CB-CD4/HTLV-1 cells, HBZ stably resides in the cytoplasm or shuttles between cytoplasm and nucleus, the cells were treated with Leptomycin B (LMB), an inhibitor of CRM-1-dependent nuclear export, and analyzed by immunofluorescence and confocal microscopy. Results clearly showed that LMB treatment did not affect HBZ cytoplasmic localization in CB-CD4/HTLV-1 T cells (Figure 11A, HBZ, top panel versus bottom panel). Interestingly, cytoplasmic localization of endogenous Tax-1 in CB-CD4/HTLV-1 cells was also not modified by the presence of LMB (Figure 11A, Tax-1, upper panel versus lower panel). This finding was at variance with the results observed in the Tax-1-transfected 293T cell line in which Tax-1 was shuttling to the nucleus as assessed by a similar LMB treatment (Forlani et al., 2016a). The efficacy of LMB treatment was shown by the nuclear accumulation of p65/RelA that, in the absence of LMB treatment, was mostly localized in the cytoplasm (Figure 11B, +LMB). From these results, we conclude that in CB-CD4/HTLV-1 cells, as in HAM/TSP patients, HBZ is specifically confined to the cytoplasm and does not translocate to the nucleus.

## Discussion

Although many advances have been obtained on the molecular and cellular mechanisms underlining the pathogenesis of infection by HTLV-1, the first described human oncogenic retrovirus (Poiesz et al., 1980), the intimate mechanisms associated to the onset of the two major diseases associated to HTLV-1 infection, namely, acute T cell leukemia-lymphoma (ATL) and tropical spastic paraparesis (HAM/TSP), are still not fully understood. In particular, the reason why some HTLV-1-infected patients develop HAM/TSP and others progress toward the neoplastic state is largely elusive.

Two viral products, Tax-1 and HBZ, are certainly involved in the pathogenesis of ATL but most likely with distinct mechanism. Tax-1 is thought to be more important in the triggering than in the maintenance of oncogenic transformation for various actions on the homeostasis of the cell initially infected by HTLV-1, particularly in the constitutive activation of the NF-kB pathway (Grassmann et al., 2005). Indeed, Tax-1 expression is not present in 40% of leukemic patients (Takeda et al., 2004). On the other hand, HBZ is persistently present in ATL and thus it is believed to play a major role in the maintenance of a neoplastic process (Matsuoka and Jeang, 2011). This is further complicated by the observation that Tax-1 and HBZ seem to have several opposing functions on specific cellular pathways involved in the control of cellular activation and proliferation (Matsuoka and Yasunaga, 2013). Mechanisms associated with the neuroinflammatory pathology of HAM/TSP depend on chronic activation of cellular immunity and damage induced by the immune response to the nervous system (Matsuura et al., 2010; Yamano and Sato, 2012; Enose-Akahata et al., 2017). The role of Tax-1 and HBZ seems to be less understood, at least in terms of recognition by immune cells and consequent outcome of protection versus immunopathology (Suemori et al., 2009; Macnamara et al., 2010; Hilburn et al., 2011).

All these events require a precise and more defined analysis of important parameters related to these viral proteins: their temporal expression, their relative amount of expression during the various phases of infection and related pathology, and, importantly, their relative subcellular distribution during the life history of infection and evolution toward distinct pathologies.

To address these issues, we have recently undertaken studies of expression, quantification, and subcellular distribution of HBZ that have been hampered in the past by the lack of suitable reagents to detect and quantify the endogenous viral protein. We have recently demonstrated for the first time that HBZ protein, previously believed as an exclusive nuclear protein, is indeed expressed in the cytoplasm of PBMCs of HAM/TSP patients (Baratella et al., 2017b), in contrast with the predominant nuclear expression in ATL cells (Raval et al., 2015). No careful studies have been reported on the expression of HBZ protein in AC.

Here, we have analyzed in detail the expression and subcellular distribution of HBZ and Tax in a cohort of AC and HAM/TSP patients. Several important findings were observed.

The confocal microscopy analysis performed on PBMCs of AC showed that HBZ protein expression could be detected in these cells, although not in all infected individuals and at a reduced number as compared to HAM/TSP. Most importantly, we show for the first time that in AC, HBZ is localized exclusively in the cytoplasm.

Cytoplasmic HBZ appears to be distributed in dots resembling the nuclear dots found in ATL cells. Thus, in the natural history HTLV-1 infection, expression of HBZ protein is primarily confined to the cytoplasm and, depending on the differential progression toward HTLV-1-associated diseases, HBZ remains cytoplasmic in HAM/TSP patients. Importantly, the increase in HTLV-1 PVL in these patients is paralleled by the increase in the number of HBZ-positive cells. By contrast, HBZ expression is more associated with the nucleus of ATL patients (Raval et al., 2015), strongly suggesting that the evolution of HTLV-1 infection toward the leukemic state is marked by the migration of HBZ to the nucleus. Within this context, recent studies have reported that HBZ-transfected cell lines, including the T cell line Jurkat, may partially segregate HBZ in the cytoplasm as a result of interaction with THEMIS (Kinosada et al., 2017), a protein expressed exclusively in T cells (Brockmeyer et al., 2011; Fu et al., 2013). However, confocal microscopy analysis of PBMCs of AC and HAM/TSP patients described in this paper shows that the expression of THEMIS is not necessary for the expression and cytoplasmic localization of endogenous HBZ. Thus, whether the distinct subcellular localization of HBZ observed in ATL as compared to AC and HAM/TSP is due to an active migration of HBZ to the nucleus or to a passive translocation due to the loss or altered expression of THEMIS remains to be elucidated. This does not exclude the idea that different cytoplasmic anchoring molecules or molecular complexes may also be involved in the cytoplasmic localization of HBZ in AC and in HAM/TSP patients (see also discussion below).

In PBMCs of AC, the Tax-1 protein was expressed in a discrete number of cells and preferentially localized in the cytoplasm. Interestingly, the expression of Tax-1 was found to be largely uncoupled to that of HBZ, and in this respect, these findings were similar to those previously reported for HAM/TSP patients (Baratella et al., 2017b). The biological basis of the mutually exclusive expression of either HBZ or Tax-1 in the same cell in AC and HAM/TSP patients is still unknown and certainly will be the focus of our future investigation.

In this study, we have significantly expanded the number of HAM/TSP patients. We confirmed that HBZ is a cytoplasmic protein, and we showed for the first time that it is expressed in a higher number of PBMCs as compared to AC. This was paralleled by a similar higher number of PBMCs expressing Tax-1, and, as mentioned above, the increase in number of cells expressing HBZ and Tax-1 correlated often, but not always, with the higher PVL found in these patients. A noticeable exception was the PH2116 AC displaying a very high PVL (19.16) and only 2 and 0% of HBZ and Tax-1 positive cells, respectively. Of interest is the observation that this AC with an uncommonly high PVL was the spouse of a HAM/TSP patient and is clinically being followed for any signs and symptoms of neurological disease.

In line with our previous findings (Baratella et al., 2017a,b), additional confocal microscopy analysis of PBMCs of AC and HAM/TSP patients clearly showed that the major, if not the exclusive, HBZ+ cell subpopulation was represented by CD4+ cells. Conversely, only a very limited number of HBZ-positive cells were found in the CD8+ T cell compartment, reinforcing the idea that in HAM/TSP as well as in AC, CD8+ T cell subpopulation is not a primary target of the HTLV-1 virus.

Although CD4+/CD25+ Tregs can be infected by HTLV-1 and HAM/TSP patients have been shown to have a high number of CD4+/CD25+ Tregs with impaired function and higher HBZ mRNA levels (Araya et al., 2011; Enose-Akahata et al., 2018), the results presented here indicate that in HAM/TSP patients, HBZ protein can be detected in CD4+ T cells not displaying the classical phenotype of Treg cells. Thus, the possible uncoupling of HBZ mRNA and protein expression in CD4+CD25+ Tregs as well as in other CD4+ T cell subpopulations in both AC and HAM/TSP patients (Yamano et al., 2009; Leal et al., 2013; Araya et al., 2014; Billman et al., 2017) certainly requires further investigation.

Further studies by confocal and biochemical analysis conducted on CB-CD4/HTLV-1, a T cell line established from an HAM/TSP patient (Ozden et al., 2004), confirmed that HBZ protein resides exclusively in the cytoplasm. Tax-1, expressed in 20% of CB-CD4/HTLV-1 cells, also localized mostly in the cytoplasm. The CB-CD4/HTLV-1 cell line was instrumental to unambiguously show that HBZ does not shuttle between the cytoplasm and nucleus at least in a CRM1-dependent manner as assessed by LMB treatment. These data, along with previous data from our group (Baratella et al., 2017b), give support to the idea that in AC and in HAM/TSP patients, HBZ may be actively retained in the cytoplasm (Baratella et al., 2017a).

In conclusion, we believe that the data presented in this research add new relevant information for a better definition of the pathological states associated with HTLV-1 infection, particularly in relation to the distinct subcellular expression of HBZ in the different pathological contests and related pathologies. Whether HBZ cytoplasmic and nuclear localization in the natural history of HTLV-1 infection represents a marker of infection or is part of the mechanism governing the evolution toward HAM/TSP or ATL is the challenge for future investigation.

## Author Contributions

GF, MB, and RA conceived the work. GF, MB, and AT performed the experiments. All authors wrote, revised, and approved the final manuscript.

## Conflict of Interest Statement

The authors declare that the research was conducted in the absence of any commercial or financial relationships that could be construed as a potential conflict of interest.
